# Influence of Oxidative Stress on Biocontrol Activity of *Cryptococcus laurentii* against Blue Mold on Peach Fruit

**DOI:** 10.3389/fmicb.2017.00151

**Published:** 2017-02-02

**Authors:** Zhanquan Zhang, Jian Chen, Boqiang Li, Chang He, Yong Chen, Shiping Tian

**Affiliations:** ^1^Key Laboratory of Plant Resources, Institute of Botany, Chinese Academy of SciencesBeijing, China; ^2^College of Life Sciences, University of Chinese Academy of SciencesBeijing, China

**Keywords:** *Cryptococcus laurentii*, biocontrol, oxidative stress, apoptosis, antioxidant systems, glutathione

## Abstract

The limitations of chemical fungicides for the control of postharvest diseases have recently become more apparent. The utilization of antagonistic microorganisms is a promising alternative to that of fungicides to control postharvest decay. In previous studies, the antagonistic yeast *Cryptococcus laurentii* has shown excellent effects of biocontrol and great potential for practical application. Adverse conditions, such as oxidative stress, limit the practical application of antagonistic yeast. In this study, we investigated the oxidative stress tolerance of *C. laurentii* and the associated mechanisms. The results indicated that exogenous oxidative stress has a significant effect on the viability and biocontrol efficiency of *C. laurentii*. H_2_O_2_-induced oxidative stress led to the accumulation of reactive oxygen species. The results of flow cytometric analysis suggested that apoptosis is responsible for the reduced survival rate of *C. laurentii* under oxidative stress. Using tests of antioxidant activity, we found that *C. laurentii* could employ enzymatic systems to resist exogenous oxidative stress. The addition of exogenous glutathione, a non-enzymatic antioxidant, to the media can significantly enhance oxidative tolerance and biocontrol efficiency of *C. laurentii*.

## Introduction

Postharvest diseases of fruits and vegetables cause considerable economic losses worldwide, and account for more than 25% of total production in developed countries and more than 50% in developing countries (Nunes, 2012). The application of chemical fungicides is currently the primary means of controlling postharvest disease. Nevertheless, the excessive use of fungicides has led to several negative effects, e.g., drug resistance of pathogens, environmental pollution, and the subsequent harm to human health (Janisiewicz and Korsten, 2002; Droby et al., 2009; Jamalizadeh et al., 2011). Therefore, the quest for safe and effective alternatives to fungicides is crucial. Antagonistic yeasts, such as *Cryptococcus laurentii*, *Rhodotorula glutinis*, and *Pichia membranifaciens*, have been exploited as promising alternatives to synthetic fungicides, and have been gradually receiving considerable attention (Fan and Tian, 2000; Qin et al., 2003, 2004; Li and Tian, 2006).

During their application, biocontrol agents are subjected to many adverse stresses that affect their survival and performance (Sui et al., 2015). Yeasts are commonly subjected to oxidative stress (Li and Tian, 2007; Macarisin et al., 2010). After pathogenic attack in the host, an oxidative burst, which is associated with increased levels of H_2_O_2_ and O_2_^-^, can be generated in the area surrounding the infection site, and serves as an early resistance response to pathogenic invasion (Segal, 2005; Temme and Tudzynski, 2009). Furthermore, antagonistic yeasts could also act as an elicitor that triggers ROS signaling in host tissue and thereby activates host defenses (Chan and Tian, 2006; Xu et al., 2008; Macarisin et al., 2010). Excessive ROS can affect the viability and biocontrol efficacy of antagonistic yeasts. However, the ability of antagonistic yeasts to withstand oxidative stress varies among different species. Liu et al. (2011a,b, 2012) examined the responses of *Metschnikowia fructicola*, *Candida oleophila*, and *Cystofilobasidium infirmominiatum* to oxidative stress. They found that *C. infirmominiatum* was sensitive and *M. fructicola* was relatively tolerant to oxidative stress. The antagonistic yeast *C. laurentii* has been widely studied and has shown excellent biocontrol efficacy against many postharvest diseases of apples, strawberries, mangoes, and sweet cherries (Tian et al., 2004; Bautista-Rosales et al., 2014; Navarta et al., 2014; Zhang et al., 2015). A previous study has indicated that oxidative stress tolerance of an antagonistic yeast species is closely associated with its biocontrol performance in postharvest application (Castoria et al., 2003). Although many studies have reported on oxidative stress resistance of antagonistic yeasts, further discovery regarding the mechanisms of action by which oxidative stress regulates their viability and biocontrol efficacy remain unknown.

The present study aimed to evaluate the tolerance of *C. laurentii* to oxidative stress and elucidate the antioxidative mechanism. Moreover, the mechanisms by which oxidative stress is used to regulate survival and biocontrol efficacy of *C. laurentii* were investigated, using flow cytometric analysis. Methods to improve oxidative stress resistance and biocontrol performance were also exploited.

## Materials and Methods

### Yeast and Pathogens

*Cryptococcus laurentii* was isolated from the surfaces of apple fruits in a previous experiment (Qin et al., 2004) and grown in YPD broth (10 g yeast extract, 20 g peptone, and 20 g dextrose in 1 L water). Yeast cultures with an initial concentration of 1 × 10^5^ cells/mL were incubated at 26°C on a rotary shaker at 200 rpm for 17 h to reach the mid-log phase. *Penicillium expansum* was isolated from naturally infected apple fruits. It was routinely cultured on potato dextrose agar plates for 14 days at 25°C. Fungal spores were harvested by flooding the surface of the culture with sterile distilled water, followed by filtration through four layers of sterile cheesecloth. The number of spores in the resulting suspension was calculated using a hemocytometer. Before inoculation, the spore concentration in sterile distilled water was adjusted to 1 × 10^4^/mL.

### Fruit

Peach fruits (*Prunus persica* L. Batsch) at commercial maturity were harvested from an orchard in Beijing and immediately transported to the laboratory. Fruits without blemishes or rot were selected based on uniformity of size. Selected fruits were surface-disinfected with 2% (v/v) sodium hypochlorite for 2 min, rinsed with tap water, and air-dried prior to further use.

### Oxidative Stress Tolerance Assays

The median lethal concentration of H_2_O_2_ for *C. laurentii* was determined according to the methods of Chen et al. (2015). Cells in the mid-log phase were obtained by centrifugation. After being washed twice with sterile distilled water, yeast cells were resuspended in fresh YPD medium to a final concentration of 5 × 10^7^ cells/mL. H_2_O_2_ was added to each yeast culture to final concentrations of 0, 100, 200, 300, and 400 mM. Following incubation for 90 min (150 rpm, 26°C), yeast cells of each sample were collected and adjusted to 1 × 10^6^ cells/mL. To analyze survival rates, a 50 μL yeast sample was spread on a YPD solid plate. The plates were subsequently observed under a light microscope (Carl Zeiss, Oberkochen, Germany). The effects of treatment time with H_2_O_2_ on yeast viability were determined using a plate assay according to the methods of Liu et al. (2011b). Yeast cell viability was expressed as a percentage of the colony number following H_2_O_2_ treatment, relative to that without treatment. For each treatment, there were three replicates and the experiment was performed twice.

### Detection of Intracellular ROS

Intracellular ROS was detected using a 10 μM 2′,7′-dichlorodihydrofluorescein diacetate (DCFH-DA) oxidant-sensitive probe (Molecular Probes, Eugene, OR, USA). DCFH-DA was added to the yeast suspension and incubated in the dark at 37°C for 30 min. After being washed twice with PBS, yeast cells were examined under a microscope (Zeiss Axioskop, Oberkochen, Germany) using a 485-nm excitation and 530-nm emission filter combination. Three independent experiments were performed.

The fluorescence intensity of *C. laurentii* cells was determined using a fluorescence microplate reader (Synergy H4, BioTek, Winooski, VT, USA). Yeast samples were washed twice with *N*-2-hydroxyethylpiperazine-*N*-2’-ethanesulfonic acid (HEPES) buffer (pH 7.0) and incubated with 10 μM DCFH-DA at 37°C for 30 min. The samples were then washed twice with HEPES buffer and diluted to an optical density (OD) at 600 nm of 1.4. Yeast samples (200 μL/well) were then added to a 96-well dark microplate, and fluorescence was analyzed using a fluorescence microplate reader with an excitation wavelength of 492 nm and an emission wavelength of 527 nm. Three replicate wells were analyzed for each treatment, and the experiment was performed twice.

### Biocontrol Analysis of *C. laurentii*

Biocontrol performance of *C. laurentii* against *P. expansum* was determined on peach fruits. *C. laurentii* cells at mid-log phase were either treated with 300 mM H_2_O_2_ for 90 min as described above or left untreated. Peach fruits were punctured at the equatorial line (three wounds per fruit) using a sterile nail, and first inoculated with 10 μL *C. laurentii* cell suspension (5 × 10^7^ cells/mL) and then with 10 μL *P. expansum* spore suspension (1 × 10^4^ spores/mL). Treated fruits were placed in plastic boxes. Each tray was enclosed with a polyethylene bag to maintain high humidity (about 95% relative humidity), and stored at 25°C. Disease incidence and lesion diameters of the fruits were recorded after 3, 4, and 5 days. Each treatment comprised three replicates with ten fruits per replicate, and the experiment was performed twice.

### Analysis of Apoptosis of *C. laurentii* Cells under Oxidative Stress

To discriminate between viable, necrotic, and apoptotic cells, flow cytometric measurements of Hoechst 33342/PI double stained yeast cells were carried out using a MoFlo XDP Cell Sorter (Beckman Coulter, Brea, CA, USA). Stained cells were analyzed using laser-based flow cytometry systems. Yeast cells at the mid-log phase were collected and treated with 300 mM H_2_O_2_ for 90 min. After being washed twice with PBS, the treated and untreated yeast cells were incubated with 10 μg/mL PI and 5 μg/mL Hoechst 33342 for 20 min in the dark. PI-positive yeast cells indicated damaged plasma membranes and the presence of necrotic cells. PI-negative and Hoechst 33342-positive yeast cells were considered apoptotic (Hong et al., 2007). The cell density of each sample was maintained at approximately 1 × 10^6^ cells/mL. The sample flow rate was 700 cells/s. A total of 20,000 cells were measured for each sample.

### Assays of CAT and SOD Activity

*Rhodotorula glutinis*, an oxidative stress-sensitive yeast, was used as a positive control in the analysis of antioxidant systems. For the enzyme activity assay, yeast cells were collected by centrifugation at specific intervals (0, 0.5, 1, 2, and 3 h) after being treated with a moderately lethal concentration of H_2_O_2_ and washed twice with PBS. The yeast cells were disintegrated with glass beads through vibration on a vortex mixer. SOD and CAT were extracted with 50 mM PBS (pH 7.0, 1 mM EDTA, and 2 mM phenylmethanesulfonyl fluoride). The reaction mixture (3 mL) with SOD contained 50 mM PBS, 13 mM methionine, 75 μM nitroblue tetrazolium, 10 μM EDTA, 2 μM riboflavin, and 50 μL enzyme extract. The mixtures were illuminated by light (4000 lx) for 20 min, and the absorbance was determined at 560 nm. Identical solutions held in the dark served as blanks. One unit of SOD activity was defined as the amount of enzyme causing 50% inhibition in the nitroblue tetrazolium reduction. The reaction mixture (1.5 mL) with CAT consisted of 1.4 mL H_2_O_2_ (40 mM) and 100 μL enzyme extract. The decomposition of H_2_O_2_ was determined based on the decline in absorbance at 240 nm. One unit of CAT activity was defined as the decomposition of 1 μM H_2_O_2_ per min. The activity of both enzymes was expressed as U/mg protein.

### Assays of Total Glutathione

Extracts for the total glutathione assay were prepared according to the methods of Ellman (1959), with minor modifications. Cells of *R. glutinis* and *C. laurentii* at the mid-log phase were collected and treated with 30 and 300 mM H_2_O_2_ for 90 min, respectively. The cells were then harvested by centrifugation at 8000 × *g* for 3 min, washed three times with sterile distilled water, resuspended in PBS (pH 7.0), and extracted by vortexing with glass beads. The extracts were used for the total glutathione assay. Total glutathione content was determined using the GSH and GSSG Assay Kit S0053 (Beyotime, Shanghai, China) and 5, 5′-dithio-bis-nitrobenzoic acid. GSSG was reduced to GSH by glutathione reductase and NADPH. The absorbance was monitored at 412 nm and the results were expressed as mM/g.

### Exogenous GSH Treatment

The effects of exogenous GSH on yeast cell viability following H_2_O_2_ treatment were determined according to the methods of Liu et al. (2011b), with slight modifications. Yeast cells with an initial concentration of 1 × 10^5^ cells/mL were supplemented with GSH to yield final concentrations of 1 and 10 mM. After overnight cultivation, yeast cells at the mid-log phase were harvested by centrifugation at 8000 × *g* for 3 min and washed three times with fresh YPD to remove any residual GSH. Yeast cells were then resuspended in fresh YPD medium to a final concentration of 5 × 10^7^ cells/mL, and *C. laurentii* cells were treated with 300 mM H_2_O_2_ for 90 min. Cell viability was evaluated by the aforementioned methods.

### Statistical Analysis

All statistical analyses were performed using the SPSS version 13 software (SPSS Inc., Chicago, IL, USA). Data were analyzed using one-way ANOVA, and comparisons between means were performed using the Duncan’s multiple range test. Differences at *P* < 0.05 were considered significant.

## Results

### Survival of *C. laurentii* under H_2_O_2_-Induced Oxidative Stress

**FIGURE 1:**
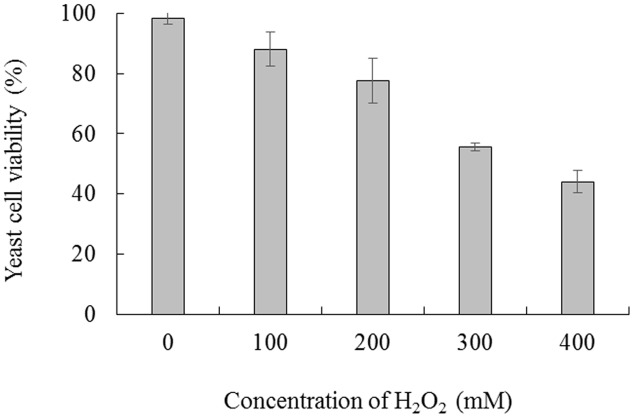
**Viability of *Cryptococcus laurentii* under oxidative stress.** *C. laurentii* cells were treated with a series of concentrations of H_2_O_2_ over 90 min. Vertical bars represent standard errors of the mean.

**FIGURE 2:**
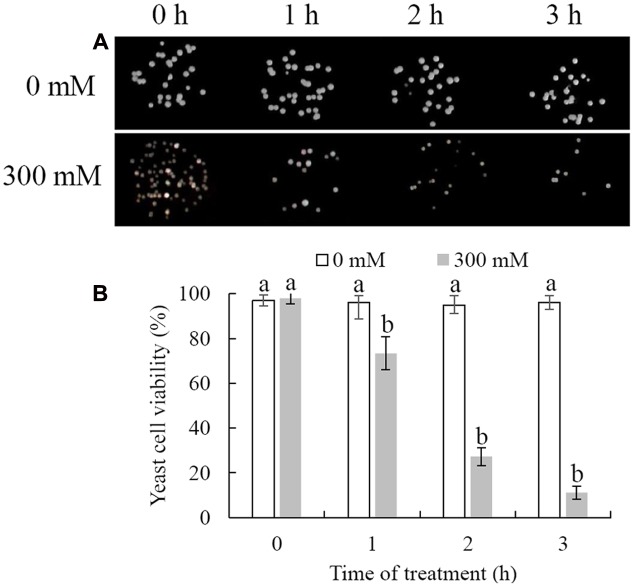
**Survival of *Cryptococcus laurentii* following treatment with 300 mM H_2_O_2_.** **(A)** Spotting assay of viability of *C. laurentii* following treatment with 300 mM H_2_O_2_. **(B)** Survival rate of *C. laurentii* following treatment with 300 mM H_2_O_2_. Vertical bars represent standard errors of the mean. Columns with different letters indicate significant differences (*P* < 0.05).

Cell viability was measured following exposure of *C. laurentii* to increasing doses of H_2_O_2_ (ranging from 0 to 400 mM) and treatment intervals in YPD liquid media. Oxidative stress induced by H_2_O_2_ significantly inhibited cell viability in a dose- and time-dependent manner, and exposure to 300 mM H_2_O_2_ over 90 min was moderately lethal to cells (about 50% inhibitory) (**Figures 1** and **2**). Based on these results, 300 mM H_2_O_2_ over 90 min was selected as the appropriate concentration and interval to promote oxidative stress for subsequent studies.

### Measurement of ROS Production

**FIGURE 3:**
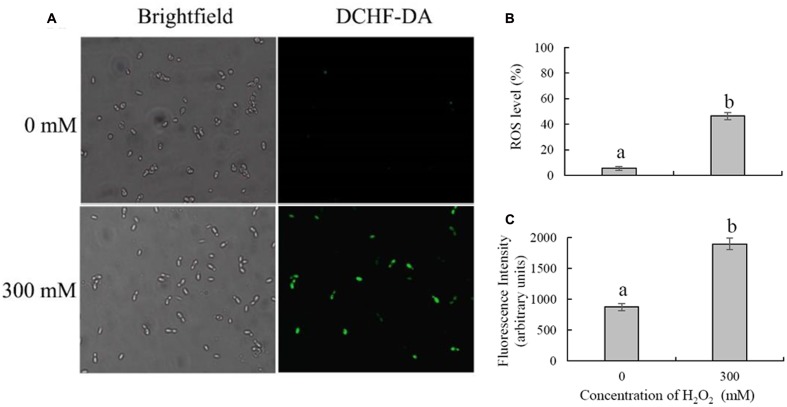
**Detection of intracellular ROS in *Cryptococcus laurentii* following treatment with and without exogenous H_2_O_2_.** **(A)** Microscopic images of *C. laurentii* cells stained with 2′,7′-dichlorodihydrofluorescein diacetate (DCFH-DA). **(B)** Percentage of *C. laurentii* cells exhibiting visible ROS accumulation. **(C)** Statistical analysis of the fluorescence intensity of *C. laurentii* cells. Vertical bars represent standard errors of the mean of three independent experiments. Columns with different letters indicate significant differences (*P* < 0.05).

Intracellular ROS production was measured by detection of fluorescence by DCFH-DA, which could be converted to highly fluorescent dichlorofluorescein in the presence of intracellular ROS. The results showed that H_2_O_2_ treatment could significantly induce the accumulation of intracellular ROS in *C. laurentii* (**Figure 3A**). Under conditions of oxidative stress induced by 300 mM H_2_O_2_, the percentage of ROS-positive cells was 46.6%. In contrast, only 5.6% of *C. laurentii* cells that were not subjected to exogenous oxidative stress showed visible ROS accumulation (**Figure 3B**). The results obtained in the analysis of fluorescence intensity were consistent with these findings. Treatment with 300 mM H_2_O_2_ significantly increased the intensity of dichlorofluorescein fluorescence in *C. laurentii* cells (**Figure 3C**).

### Biocontrol Efficacy of *C. laurentii*

**FIGURE 4:**
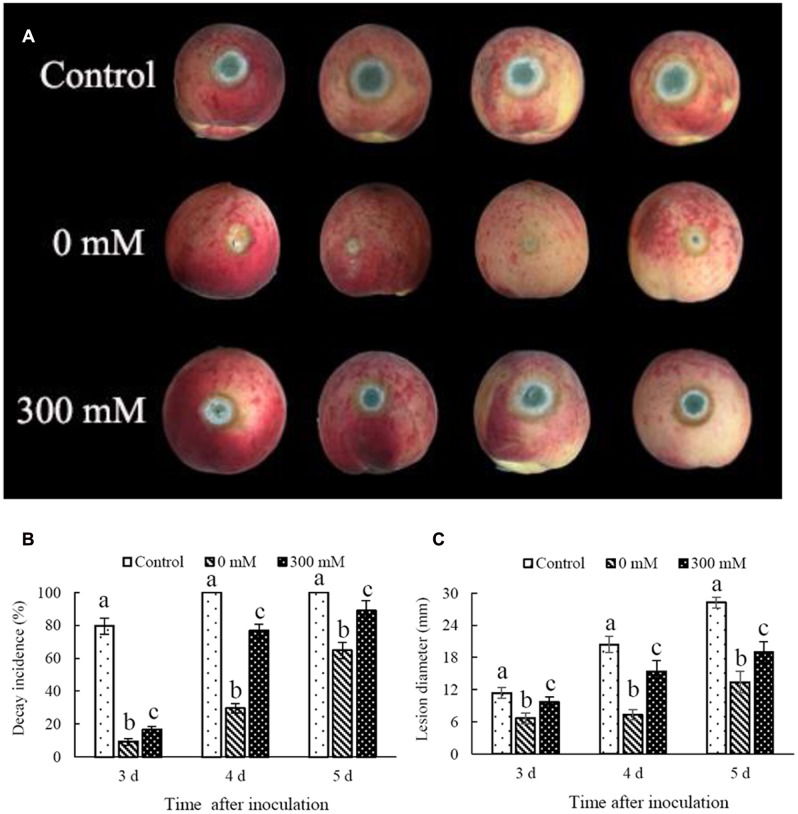
**Effect of oxidative stress on biocontrol efficiency of *Cryptococcus laurentii* against *Penicillium expansum*.** **(A)** Biocontrol performance of H_2_O_2_-treated *C. laurentii* cells and non-treated *C. laurentii* cells against *P. expansum* on peach fruits (5 days post inoculation). **(B)** Statistical analysis of decay incidence on peach fruits 3, 4, and 5 days post inoculation. **(C)** Statistical analysis of lesion diameters on peach fruits 3, 4, and 5 days post inoculation. Vertical bars represent standard errors of the mean. Columns with different letters indicate significant differences (*P* < 0.05).

In comparison with the control, non-H_2_O_2_-treated *C. laurentii* cells effectively inhibited postharvest decay caused by *P. expansum* on peach fruits (**Figure 4A**). Notably, the addition of 300 mM H_2_O_2_ significantly reduced biocontrol activity of *C. laurentii*. After 4 days, disease incidence in peach fruits treated with *C. laurentii* cells that had not been subjected to oxidative stress was 30%; whereas disease incidence in H_2_O_2_-treated samples was as high as 76% (**Figure 4B**). Moreover, the efficiency of *C. laurentii* cells in inhibiting lesion expansion was also significantly suppressed under exogenous oxidative stress (**Figure 4C**). These results suggest that exogenous oxidative stress could exert significant influence on biocontrol efficiency of *C. laurentii*. Thus, improving oxidative stress tolerance in *C. laurentii* is essential to enhancing its biocontrol efficiency.

### Cell Membrane Integrity and Apoptosis

**FIGURE 5:**
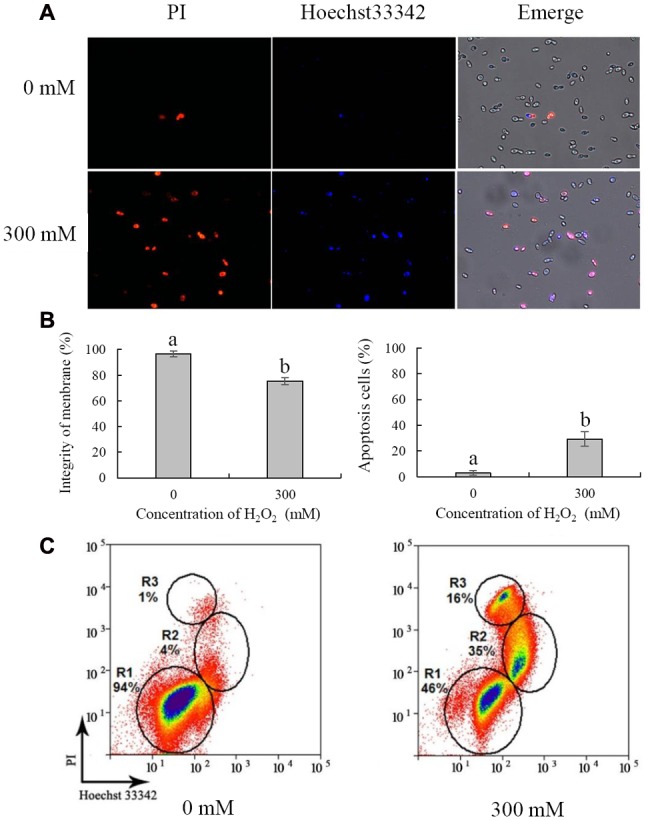
**Necrosis and apoptosis analysis of *Cryptococcus laurentii* cells under oxidative stress.** **(A)** Fluorescence microscopic image of *C. laurentii* cells treated with and without exogenous H_2_O_2_ and double stained with PI/Hoechst 33342. **(B)** Percentage of *C. laurentii* cells with plasma membrane integrity and percentage of apoptotic cells. **(C)** Flow cytometric analysis. R1 represents viable cells, R2 represents apoptotic cells, and R3 represents necrotic cells. Vertical bars represent standard errors of the means of three independent experiments. Columns with different letters indicate significant differences (*P* < 0.05).

Necrosis and apoptosis of *C. laurentii* cells were demonstrated using Hoechst 33342 and PI staining. Necrotic yeast cells with a damaged plasma membrane were detectable, based on their high red fluorescence due to PI uptake (**Figure 5A**). Following exposure to 300 mM H_2_O_2_ for 90 min, the integrity of the plasma membrane of *C. laurentii* cells was reduced to 75%, and the number of stained apoptotic yeast cells was increased to 29% (**Figure 5B**). However, damage to the plasma membrane and apoptosis were not significant in *C. laurentii* cells that had not been subjected to H_2_O_2_ treatment (**Figures 5A,B**).

For further analysis, necrosis and apoptosis in *C. laurentii* cells exposed to H_2_O_2_ were examined by flow cytometric analysis. On the dot plots, each cell is represented by a single dot (**Figure 5C**). When cells were stained with a combination of Hoechst 33342 and PI, three cell populations were observed: viable (R1), apoptotic (R2), and necrotic (R3) (**Figure 5C**). In the absence of H_2_O_2_-induced oxidative stress, the vast majority of *C. laurentii* cells was viable; however, following treatment with 300 mM H_2_O_2_, the percentage of viable cells was reduced to 46%, and the percentage of apoptotic and necrotic cells was 35 and 16%, respectively. These results are consistent with those of the survival analysis of *C. laurentii* under oxidative stress. These findings suggest that exogenous oxidative stress induced by 300 mM H_2_O_2_ could lead to apoptosis of *C. laurentii* cells that is primarily responsible for the decline in viability under oxidative stress.

### Antioxidant Enzyme and Total Glutathione Assays

**FIGURE 6:**
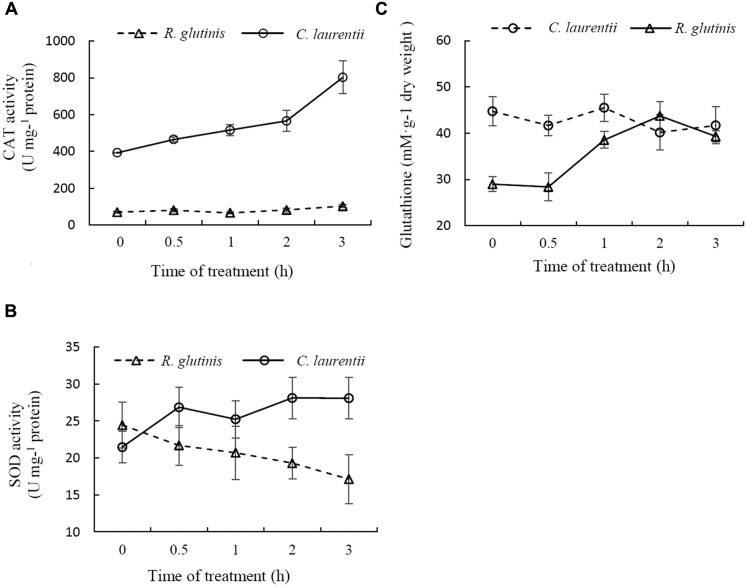
**Comparison between antioxidative reactions of *Cryptococcus laurentii* and *Rhodotorula glutinis* following treatment with moderately lethal concentrations of H_2_O_2_.** **(A)** Determination of CAT activities of *C. laurentii* and *R. glutinis* cells following treatment with 300 mM and 30 mM H_2_O_2_, respectively. **(B)** Determination of SOD activities of *C. laurentii* and *R. glutinis* cells following treatment with 300 mM and 30 mM H_2_O_2_, respectively. **(C)** Determination of glutathione activity of *C. laurentii* and *R. glutinis* cells following treatment with 300 mM and 30 mM H_2_O_2_, respectively. Vertical bars represent standard errors of the means of three independent experiments.

To resist the effects of oxidative stress and maintain cellular homeostasis, cells have evolved two sophisticated antioxidant systems, the enzymatic (e.g., CAT and SOD) and non-enzymatic antioxidant (e.g., glutathione and vitamins) defense systems. CAT and SOD are two major antioxidant enzymes involved in the enzymatic antioxidant defense system. Previous studies have shown that treatment with 30 mM H_2_O_2_ for 90 min has a moderately lethal effect on *R. glutinis* cells (about 50% inhibitory). In contrast, the same concentration of H_2_O_2_ has no significant effects on the viability of *C. laurentii* (data not shown), indicating that in comparison to *R. glutinis*, *C. laurentii* shows greater resistance to exogenous oxidative stress. To determine the reasons for this higher oxidative tolerance of *C. laurentii*, the enzyme activities of CAT, SODe and total glutathione of *R. glutinis* and *C. laurentii* under moderately lethal oxidative stress (*R. glutinis*: 30 mM H_2_O_2_; *C. laurentii*: 300 mM H_2_O_2_), were assessed. CAT activity in *C. laurentii* was strongly induced by H_2_O_2_ and maintained at a higher level than it was in *R. glutinis*. The activity level in *C. laurentii* was approximately eightfold that in *R. glutinis* (**Figure 6A**). Similarly, H_2_O_2_ treatment was able to induce SOD activity in *C. laurentii*. In contrast, exogenous oxidative stress showed slight inhibitory effects on SOD activity in *R. glutinis* (**Figure 6B**). These data indicate that enzymatic antioxidants are major contributors to the overall antioxidant capacity of *C. laurentii* under exogenous oxidative stress.

Glutathione also plays an important role in cellular antioxidant defenses. More than 90% of the total glutathione pool was in the reduced form (GSH) and the remainder was in the oxidized form (glutathione disulfide, GSSG). As shown in **Figure 6C**, total glutathione levels in *C. laurentii* cells remained relatively stable under H_2_O_2_-induced oxidative stress. However, this form of oxidative stress showed an obvious inductive effect on total glutathione levels in *R. glutinis*. In terms of resistance to oxidative stress, the difference between *C. laurentii* and *R. glutinis* might be dependent on the various antioxidant systems.

### GSH Treatment Improves the Viability and Biocontrol Efficiency of *C. laurentii*

**FIGURE 7:**
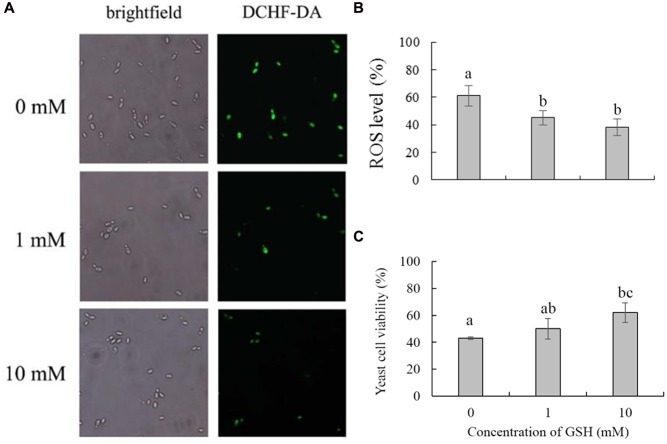
**Effects of GSH treatment on oxidative stress tolerance of *Cryptococcus laurentii*.** **(A)** ROS accumulation in *C. laurentii* cells under oxidative stress following treatment with different concentrations of GSH. Detection of intracellular ROS was facilitated by staining with DCHF2-DA. **(B)** Percentage of *C. laurentii* cells exhibiting visible ROS accumulation under oxidative stress following treatment with GSH. **(C)** Viability of *C. laurentii* cells under oxidative stress following treatment with GSH. Vertical bars represent standard errors of the means of three independent experiments. Columns with different letters indicate significant differences (*P* < 0.05).

**FIGURE 8:**
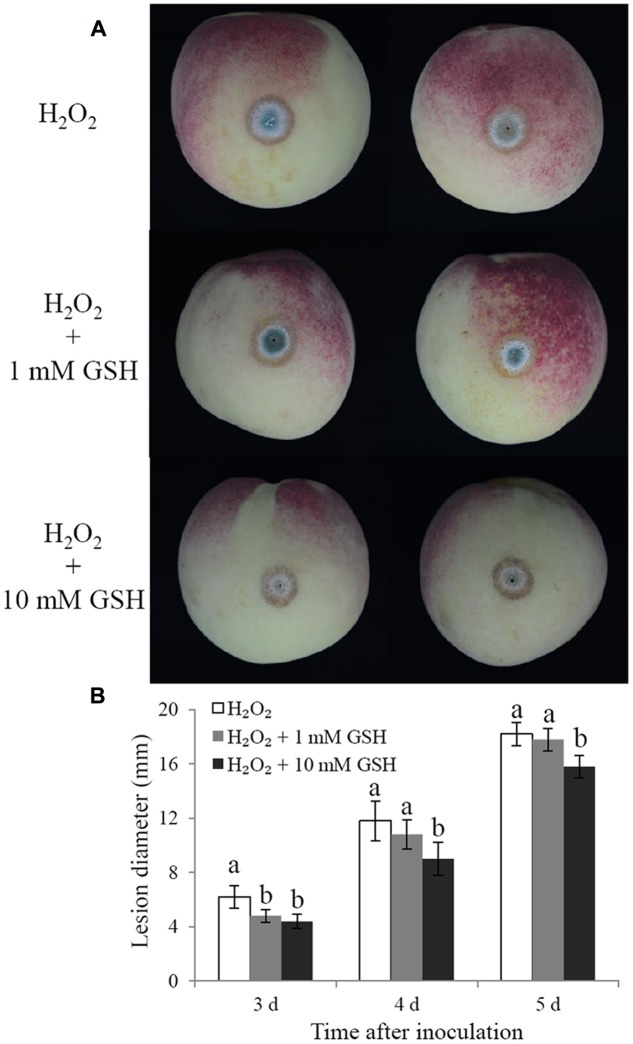
**Effects of GSH treatment on the biocontrol efficiency of *C. laurentii*.** **(A)** Signs of disease on peach fruits at 5 days post inoculation. **(B)** Statistical analysis of lesion diameters on peach fruits at 3, 4, and 5 days post inoculation. Vertical bars represent standard errors of the mean. Columns with different letters indicate significant differences (*P* < 0.05).

Glutathione is considered the main ROS scavenger in cells (Schafer and Buettner, 2001). We evaluated the effects of exogenous GSH on the cell viability and biocontrol performance of *C. laurentii* under exogenous oxidative stress. Adding GSH to the culture medium could suppress the accumulation of intracellular ROS and enhance the tolerance of *C. laurentii* to H_2_O_2_-induced oxidative stress. When treated with 10 mM GSH, the percentage of *C. laurentii* cells exhibiting visible ROS staining was reduced by 23% (**Figures 7A,B**). When 10 mM GSH was added to YPD medium, the cell viability of *C. laurentii* was improved by 19% under moderately lethal oxidative stress (**Figure 7C**). Furthermore, we validated the beneficial effects of exogenous GSH on the biocontrol efficiency of *C. laurentii* against blue mold on peach fruits. Treatment with 1 mM GSH improved biocontrol efficiency of *C. laurentii* at the earlier intervals following inoculation (**Figures 8A,B**), whereas 10 mM GSH improved biocontrol efficacy of *C. laurentii* throughout the entire experiment. On days 4 and 5 post inoculation, the lesion diameters were reduced by 24 and 13%, respectively, following treatment with 10 mM GSH (**Figures 8A,B**).

## Discussion

In this study, we investigated the influence of exogenous oxidative stress on the viability and biocontrol efficiency of *C. laurentii* and the putative mechanisms of action. Furthermore, the methods by which the oxidative resistance of *C. laurentii* can be improved were explored.

Over the past few decades, numerous laboratory studies have been conducted to evaluate the biocontrol performance of antagonistic yeasts (Droby et al., 2009; Sharma et al., 2009; Nunes, 2012; Liu et al., 2013). However, only a few yeast-based biocontrol products are presently available commercially. Various stresses in the natural environment that have significant effects on yeast viability and product stability make the use of commercial antagonistic yeasts as biocontrol agents particularly challenging (Chen et al., 2015; Sui et al., 2015). Oxidative stress is one of the major challenges posed to antagonistic yeasts in the control of pre- and postharvest diseases (Li and Tian, 2007; Macarisin et al., 2010). Enhancing the tolerance of antagonistic yeasts to oxidative stress is an effective way to improve their biocontrol ability.

The results of the present study indicate that *C. laurentii* possesses higher levels of adaptability to oxidative stress in comparison with other antagonistic yeasts, such as *R. glutinis* and *C. infirmominiatum*. Treating *C. laurentii* with 300 mM H_2_O_2_ for 90 min was moderately lethal to the cells (**Figure 1**), whereas the median lethal concentration of H_2_O_2_ for *R. glutinis* was 30 mM (Chen et al., 2015). After 20 min of incubation, the survival of *C. infirmominiatum* in 20 mM H_2_O_2_ was 23% (Liu et al., 2011b). Previous studies have indicated that competition for space and nutrients is a major factor that affects the resistance of antagonistic yeasts to postharvest fungal pathogens (Chan and Tian, 2005; Bautista-Rosales et al., 2014). These fungal pathogens infect the host tissue mainly through wounds inflicted during harvest, transportation, packinghouse operations, and storage processes (Barkai-Goland, 2001). Therefore, wound competence of yeasts is important in their mechanisms of antagonism against pathogens, even as they compete for space and nutrients. Castoria et al. (2003) suggested that resistance to oxidative stress represents a pivotal mechanism of action involved in wound competence of antagonistic yeasts, which is closely associated with their biocontrol activity. The *C. laurentii* isolate LS-28 showed higher biocontrol activity, compared with the *R. glutinis* isolate LS-11, a finding that could be attributed to the higher tolerance of *C. laurentii* to oxidative stress, as compared with *R. glutinis* (Castoria et al., 2003). In the present study, we also found that the biocontrol efficiency of *C. laurentii* was significantly suppressed under oxidative stress (**Figure 4**).

As signal molecules, ROS can regulate senescence, apoptosis, and the stress response (Perrone et al., 2008). Low concentrations of ROS can activate a variety of antioxidant systems in yeast cells, and delay cell division that relies on the transcription factors Yap1p and Msn2/4p, thereby enhancing the resistance of yeast cells to subsequent lethal stress (Collinson and Dawes, 1992; Temple et al., 2005). However, excessive oxidative stress can cause a series of injuries to cellular components, including the cell membrane, proteins, lipids, and nucleic acids, resulting in compromised cell function or loss of viability (Reverter-Branchat et al., 2004; Branduardi et al., 2007).

Apoptosis and necrosis are two common forms of cell death that are associated with the viability of yeast cells. Apoptosis is a highly regulated form of programmed cell death that is characterized by nuclear DNA fragmentation, condensed chromatin, and inversion of the plasma membrane (Madeo et al., 1999; Poljak et al., 2003). The difference between apoptosis and necrosis is mainly manifested in the integrity of the cell membrane. When apoptosis occurs, the cell membrane remains intact, whereas in the necrotic cell, the membrane is broken down. The cell dye Hoechst 33342 has strong cell membrane permeability. However, PI cannot permeate the intact cell membrane. Flow cytometric analysis combined with Hoechst 33342-PI double staining is usually used to distinguish between viable, apoptotic, and necrotic cells (Sriram et al., 1992; Vermes et al., 2000). Thus, we used this method to investigate the mechanisms whereby H_2_O_2_ causes cell death in *C. laurentii*. The results of flow cytometry suggested that exogenous oxidative stress primarily triggered apoptosis in *C. laurentii* cells, resulting in the eventual suppression of viability. This indicated that the main mechanism associated with exogenous oxidative stress on *C. laurentii* cells was both systematic and progressive.

Both enzymatic and non-enzymatic antioxidant defense systems exist in yeasts (Jamnik and Raspor, 2005). In enzymatic antioxidant defense systems, SOD and CAT are two important components (Scandalios, 1993; Lee and Lee, 2000). SOD catalyzes the superoxide radical to H_2_O_2_, and H_2_O_2_ is then converted to H_2_O and O_2_, via the action of CAT. GSH, a small antioxidant molecule that is ubiquitous in plants and animals, plays a vital role in maintaining the antioxidant status of organisms (Cnubben et al., 2001; Herouart et al., 2002). *C. laurentii* cells subjected to treatment with exogenous H_2_O_2_, mainly employ enzymatic antioxidant defense systems against oxidative stress. In contrast, the ROS-sensitive yeast strain, *R. glutinis* tends to use the non-enzymatic antioxidant glutathione to resist oxidative stress. This might explain the disparity observed between *C. laurentii* and *R. glutinis* in their tolerance to oxidative stress.

Methods for improving stress resistance of biocontrol yeasts include preadaptation to stress, physiological manipulation, and the addition of anti-stress compounds to the medium. Previous reports have shown that combining biocontrol yeasts with exogenous chemical compounds, such as calcium (Tian et al., 2002), salicylic acid (Qin et al., 2003), sodium bicarbonate (Yao et al., 2004), and trehalose (Li and Tian, 2006) are effective ways to enhance their biocontrol performance. GSH has strong antioxidant capacity, and can be easily absorbed by cells. In the present study, we first validated the application of exogenous GSH as an effective method to improve oxidative stress tolerance in *C. laurentii*. In addition, the protective effect of GSH on the biocontrol efficiency of *C. laurentii* was confirmed on altered peach fruits. These results provide us with a potential alternative to enhancing the environmental adaptability and biocontrol performance of antagonistic yeasts.

## Conclusion

We found that oxidative stress could induce apoptosis in *C. laurentii* that further leads to a reduction in cell viability and biocontrol efficiency. The enzymatic defense system might play a significant role in the antioxidative effects of *C. laurentii*. The addition of the non-enzymatic compound GSH to the culture media is an effective method to improve the oxidative stress resistance and biocontrol efficiency of *C. laurentii*.

## Author Contributions

ST conceived and designed the experiments. ZZ, JC, CH, and YC performed the experiments. ZZ analyzed the data. ZZ and BL drafted the manuscript. All authors read and approved the final manuscript.

## Conflict of Interest Statement

The authors declare that the research was conducted in the absence of any commercial or financial relationships that could be construed as a potential conflict of interest.

## Abbreviations

CAT: catalase
GSH: reduced glutathione
GSSG: oxidized glutathione
NADPH: nicotinamide adenine dinucleotide phosphate
PBS: phosphate buffered saline
PI: propidium iodide
ROS: reactive oxygen species
SOD: superoxide dismutase
YPD: yeast peptone dextrose
